# Identification of curable high-risk prostate cancer using radical prostatectomy alone: who are the good candidates for undergoing radical prostatectomy among patients with high-risk prostate cancer?

**DOI:** 10.1007/s10147-018-1272-9

**Published:** 2018-03-27

**Authors:** Kazuhiro Nagao, Hideyasu Matsuyama, Hiroaki Matsumoto, Takahito Nasu, Mitsutaka Yamamoto, Yoriaki Kamiryo, Yoshikazu Baba, Akinobu Suga, Yasuhide Tei, Satoru Yoshihiro, Akihiko Aoki, Tomoyuki Shimabukuro, Keiji Joko, Shigeru Sakano, Kimio Takai, Shiro Yamaguchi, Jumpei Akao, Seiji Kitahara, Yamaguchi Uro-Oncology Group

**Affiliations:** 1 Department of Urology, Graduate School of Medicine, Yamaguchi University, 1-1-1 Minami-Kogushi, Ube, 755-8505 Japan; 2 Department of Urology, Shuto General Hospital, 1000-1 Kogaisaku, Yanai, 742-0032 Japan; 3 Department of Urology and Nephrology, Tokuyama Central Hospital, 1-1 Takada, Shunan, 745-8522 Japan; 4 Department of Urology, Yamaguchi Grand Medical Center, 77 Osaki, Hofu, Yamaguchi 747-8511 Japan; 5 Department of Urology, Shimonoseki Saisekai Toyoura Hospital, 7-3 Kogushi, Toyoura, Shimonoseki, 759-6302 Japan; 6 Department of Urology, Yamaguchi Red Cross Hospital, 53-1 Hachimanbaba, Yamaguchi, 753-8519 Japan; 7 Department of Urology, Kanmon Medical Center, 1-1-1 Chofusotoura-cho, Shimonoseki, 752-8510 Japan; 8 Department of Urology, Shimonoseki City Hospital, 1-13-1 Koyo, Shimonoseki, 750-8520 Japan; 9 Department of Urology, Masuda Red Cross Hospital, I 103-1 Otoyoshi-chou, Masuda, 698-8501 Japan; 10 Department of Urology, Ube-kohsan Central Hospital Corp, 750 Nishikiwa, Ube, 755-0151 Japan; 11 Department of Urology, Saiseikai Yamaguchi General Hospital, 2-11 Midorimachi, Yamaguchi, 753-0078 Japan; 12 Department of Urology, Kokura Memorial Hospital, 3-2-1 Asano, Kokura-ku, Kitakyusyu, Fukuoka 802-8555 Japan; 13 Department of Urology, Saiseikai Shimonoseki General Hospital, 8-5-1 Yasuoka, Shimonoseki, 759-6603 Japan; 14 Department of Urology, Shimonoseki Medical Center, 3-3-8 Kamishinchi-cho, Shimonoseki, 750-0061 Japan; 15 Department of Urology, Ogori Daiichi General Hospital, 862-3 Ogori Shimogo, Yamaguchi, 754-0002 Japan; 16 Department of Urology, Sanyo-Onoda Municipal Hospital, 1863-1 Higashi-Takadomari, Sanyo-Onoda, Yamaguchi 756-0094 Japan

**Keywords:** Prostate cancer, High risk, Radical prostatectomy, Biochemical progression, Risk factor

## Abstract

**Background:**

Currently, there is no consensus regarding which patients with high-risk prostate cancer (PCa) would benefit the most by radical prostatectomy (RP). We aimed to identify patients with high-risk PCa who are treatable by RP alone.

**Methods:**

We retrospectively reviewed data on 315 patients with D’Amico high-risk PCa who were treated using RP without neoadjuvant or adjuvant therapy at the institutions of the Yamaguchi Uro-Oncology Group between 2009 and 2013. The primary endpoint was biochemical progression-free survival (bPFS) after RP. Risk factors for biochemical progression were extracted using the Cox proportional hazard model. We stratified the patients with high-risk PCa into 3 subgroups based on bPFS after RP using the risk factors.

**Results:**

At a median follow-up of 49.9 months, biochemical progression was observed in 20.5% of the patients. The 2- and 5-year bPFS after RP were 89.4 and 70.0%, respectively. On multivariate analysis, Gleason score (GS) at biopsy (≥ 8, HR 1.92, *p* < 0.05) and % positive core (≥ 30%, HR 2.85, *p* < 0.005) were independent predictors of biochemical progression. Patients were stratified into favorable- (0 risk factor; 117 patients), intermediate- (1 risk factor; 127 patients), and poor- (2 risk factors; 57 patients) risk groups, based on the number of predictive factors. On the Cox proportional hazard model, this risk classification model could significantly predict biochemical progression after RP (favorable-risk, HR 1.0; intermediate-risk, HR 2.26; high-risk, HR 5.03; *p* < 0.0001).

**Conclusion:**

The risk of biochemical progression of high-risk PCa after RP could be stratified by GS at biopsy (≥ 8) and % positive core (≥ 30%).

## Introduction

According to the European Association of Urology (EAU) guideline 2017, patients with high-risk prostate cancer (PCa) are reported to have increased risks of biochemical and metastatic progression and cancer-related deaths, and they need secondary treatment [1]. Nevertheless, not all patients with high-risk PCa are reported to have uniformly poor prognoses after undergoing radical prostatectomy (RP) [2]. When the tumor is not fixed to the pelvic wall or does not invade the urethral sphincter, RP is considered a reasonable first step for the treatment of selected patients [1]; however, there is no consensus regarding which patients with high-risk PCa would benefit the most from undergoing RP.

Briganti et al. reported the clinical course of surgically treated D’Amico high-risk PCa. In their study, the biochemical progression-free survival (bPFS) rate at 5 years after RP was 55.2% [3]. Additionally, when managed with non-curative intent, the cancer-specific mortality rates of patients with high-risk PCa at 10 and 15 years have been reported to be 28.8 and 35.5%, respectively [4].

Although Ploussard et al. [5] and Joniau et al. [6] stratified the patients with D’Amico high-risk PCa including the cT3 and cT4 stages who were treated with RP into 3 prognostic categories using clinical T stage (< cT3 vs cT3–4), Gleason score (GS, < 7 vs 8–10) and prostate specific antigen (PSA, ≤ 20 vs > 20 ng/ml), these studies involved patients treated with adjuvant treatments after RP. We aimed to identify patients with treatable D’Amico high-risk PCa who could be treated using RP alone, and those who should be treated using multidisciplinary approaches.

## Patients and methods

We retrospectively reviewed the medical records of 315 patients with PCa who were classified as high-risk per the D’Amico criteria, who were treated with RP without neoadjuvant or adjuvant therapy, at the 17 institutions of the Yamaguchi Uro-Oncology Group, between 2009 and 2013. This observational study was approved by the Institutional Review Board (no: H27-086) at the Yamaguchi University Hospital.

The patient characteristics are listed in Table 1. We evaluated the performance status of the patients based on Eastern Cooperative Oncology Group (ECOG) performance status. Prostate volume was estimated from the maximum transverse diameter (*D*1), the maximum anteroposterior diameter (*D*2) and the maximum longitudinal diameter (*D*3). Prostate volume was calculated using the prostate ellipse dimension theory formula (*D*1 × *D*2 × *D*3 × *π*/6). PSA density was calculated by dividing the preoperative PSA value by prostate volume. We classified the localization of prostate cancer based on the positive core in biopsy into 2 groups with or without positive specimens from the apex specimens. And, all tumors were staged based on the 2009 TNM classification system [7].

**Table 1:** Patient characteristics of the study

Characteristics	Mean (range)	
	RP	IMRT
Case number	315	100
Age (years)	68.1 (49–86)	70.2 (53–78)
ECOG performance status (0/1)	313/2	
PSA (ng/ml)	10.4 (2.9–58.7)	19.5 (4.1–45.2)
Prostate volume (ml)	29.8 (5.9–150)	26.4 (8.6–100)
PSA density	0.41 (0.04–2.38)	1.07 (0.10–2.30)
Digital rectal examination		
Normal	208	–
Abnormal	68	–
Unknown	39	–
% positive core (%)	34.1 (6.3–100)	47.6 (10.0–100)
Laterality		
Unilateral	186	–
Bilateral	126	–
Unknown	3	–
Localization		
Apex	208	–
Non-apex	66	–
Unknown	41	–
Gleason score at biopsy		
≤ 7	193	26
≥ 8	121	74
Unknown	1	0
cT		
≤ 2b	170	59
2c	145	41
Neutrophil lymphocyte ratio	2.2 (0.7–15.5)	–
Operative method		
Open	260	–
Robot	55	–
Nerve preservation		
No	263	–
Yes	52	–
Lymph node dissection		
Standard or extended	170	–
Limited	145	–
Blood loss (ml)	1103 (14–4817)	–
Gleason score at surgery		
≤ 7	167	–
≥ 8	148	–
pT		
< 3a	240	–
≥ 3a	74	–
Unknown	1	–
pN		
0	310	–
1	5	–
EPE		
–	197	–
+	72	–
Unknown	46	–
RM		
–	190	–
+	97	–
Unknown	28	–
ly		
–	259	–
+	53	–
Unknown	3	–
v		
–	283	–
+	29	–
Unknown	3	–
pn		
–	127	–
+	185	–
Unknown	3	–
PSA nadir	0.02 (0–2.79)	–
Follow-up periods (months)	49.9 (1.9–1335)	50.9 (0–125)
Biochemical (PSA) recurrence		
–	244	92
+	63	8
Unknown	8	0
Prognosis		
No evidence of disease (NED)	244	86
Alive with disease (AWD)	59	11
Other cause death	2	2
Cancer death	1	1
Unknown	9	0

The definition of D’Amico high-risk included higher PSA levels of > 20 ng/ml, higher GS ≥ 8, or clinical T2c stage. Biochemical recurrence was defined as the occurrence of serum PSA levels that were ≥ 0.2 ng/ml. In the patients in whom the PSA level did not decrease to < 0.2 ng/ml after surgery, the date of biochemical recurrence was determined as the date of surgery.

The primary endpoint of the study was bPFS after RP. Risk factors for biochemical progression were extracted using the Cox proportional hazard model. Continuous variables were dichotomized by the median value of each factor. Using the risk factors, we constructed a classification model predicting bPFS after RP.

Additionally, we retrospectively reviewed the medical records of 100 patients with D’Amico high-risk PCa who were treated with intensity modulated radiation therapy (IMRT) at the Yamaguchi University Hospital, and compared the results with those obtained from the RP cohort. The characteristics of these patients too are listed in Table 1.

Regarding the IMRT cohort, a total of 78 Gy (1.5 Gy/fraction × 52) of extra-beam radiation therapy was administered concurrently with 2-year androgen deprivation therapy (ADT). Biochemical recurrence was defined as three consecutive increases in serum PSA levels above 2 ng/ml from the nadir.

The Kaplan–Meier method and the log-rank test were used to estimate the statistical differences in bPFS. Univariate analysis was performed by Chi square test. Multivariate analysis was performed, using the Cox proportional hazards model, for the estimation of the factors predicting biochemical progression. All the reported p values were 2-sided, and all statistical analyses were performed using JMP ver. 10 (SAS Institute Inc., Cary, NC, USA).

## Results

**Fig. 1:**
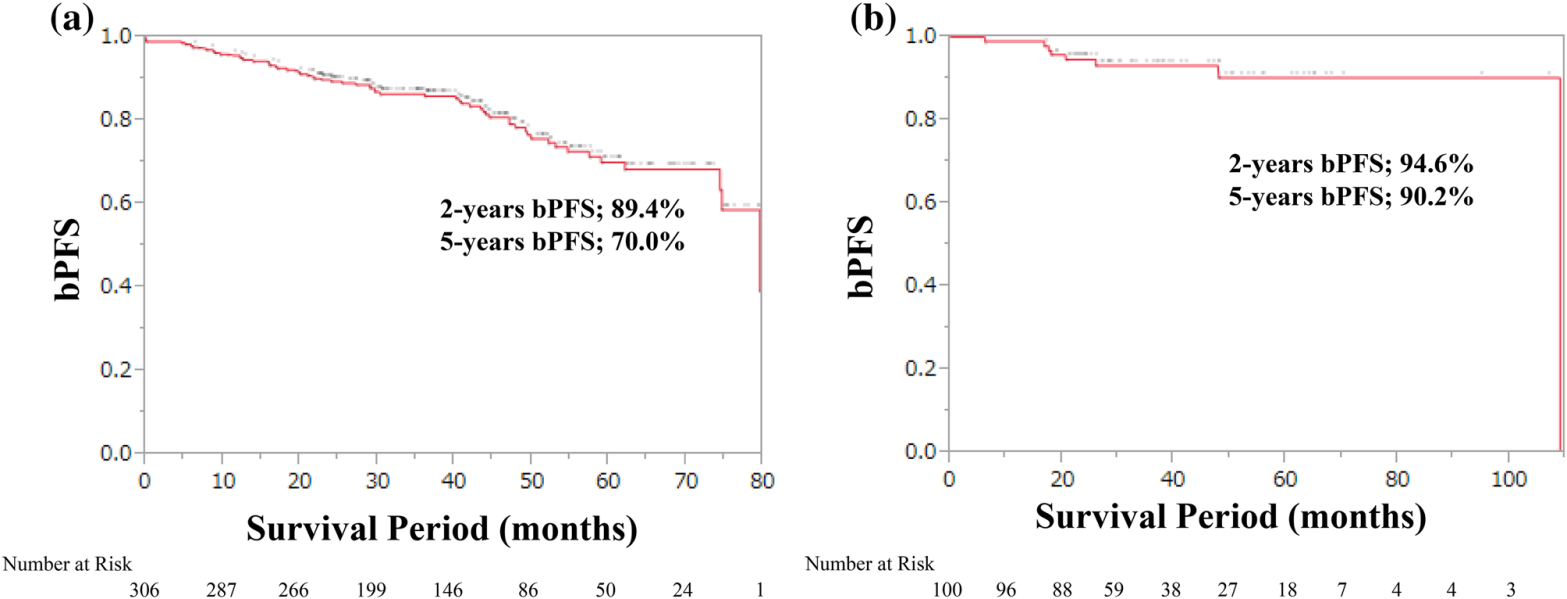
**a** Biochemical progression-free survival after radical prostatectomy (RP) for patients with D’Amico high-risk PCa. **b** Biochemical progression-free survival after intensity modulated radiation therapy (IMRT) for patients with D’Amico high-risk PCa

The characteristics of patients are listed in Table 1, and the risk factors of D’Amico high-risk PCa treated with RP are summarized in Table 2. The mean age of the patients was less than 70 years, and most of the patients had good performance status. The mean value of PSA levels at biopsy was approximately 10 ng/ml, and 24.8% patients showed high PSA values of > 20 ng/ml. More than one-third (38.5%) of the patients treated with RP had higher Gleason scores of ≥ 8 at biopsy and approximately one-half (46.0%) of the patients were at cT2c stage. After undergoing RP, the down- and up-gradings were observed in 14.6 and 45.7% of the patients, respectively. Almost one-half of the patients (53.9%) were treated with extended lymph node dissection (LND), while the rest (46.0%) were treated with limited LND. Lymph node metastasis was detected in less than 2% of the patients, and positive surgical margins (RM) were found in 30.8% of the patients. A PSA nadir of > 0.2 ng/ml after surgery was found in less than 2% of patients. At a median follow-up time of 49.9 months (range 1.9–1335), biochemical progression was observed in 20.0% of the patients. The 2- and 5-year bPFS rates after RP were 89.4 and 70.0%, respectively (Fig. 1a).

**Table 2:** Risk factors of D’Amico high-risk Pca treated by RP

D’Amic high-risk factors	RP	IMRT
Single factor		
(1) PSA ≥ 20 ng/ml	63	9
(2) Gleason score at biopsy ≥ 8	96	38
(3) cT2c	127	13
Multiple factors		
(1) + (2)	11	12
(1) + (3)	4	3
(2) + (3)	12	14
All	2	9
Unknown	1	2

In the IMRT cohort with 100 patients, the age, PSA, PSA density, GS at biopsy, and clinical stage of patients were significantly higher than the corresponding values in our RP cohort (Table 1). At a median follow-up time of 50.9 months (range 0–125), biochemical progression was observed in 8.0% patients, and 2- and 5-year bPFS were 94.6 and 90.2%, respectively (Fig. 1b).

Table 3 lists the clinicopathological parameters that may predict biochemical progression, as analyzed by univariate analysis. Per the table, among the pre-treatment factors, the PSA value at diagnosis (≥ 15 ng/ml), PSA density (≥ 0.5), GS at biopsy (≥ 8) and % positive core (≥ 30%) may be risk factors for biochemical progression after undergoing RP. In the post-operative factors, Gleason score ≥ 8 at surgery, pT ≥ 3a stage, pN1 stage, extra-prostatic extension (EPE) 1, resection margin (RM) 1, ly1, v1, pn1, and PSA nadir (≥ 0.1 ng/ml) may be significant predictive factors for biochemical progression.

**Table 3:** Predictors for biochemical recurrence after radical prostatectomy

Variables	Category	Univariate		
		HR	95% CI	*p* value
Pre-operative factors				
Age (< 65)	< 65 vs. ≥ 65 years	1.26	0.72–2.12	0.4
PSA (≥ 15)	< 15 vs. ≥ 15 ng/ml	2.25	1.27–3.84	0.0068
Prostate volume (< 30)	< 30 vs. ≥ 30 ml	1.20	0.68–2.21	0.53
PSA density (≥ 0.5)	< 0.5 vs. ≥ 0.5	2.01	1.12–3.52	0.02
Digital rectal examination (abnormal)	Abnormal vs. normal	1.73	0.97–3.01	0.06
Localization 1	Unilateral vs. bilateral lobe	1.10	0.65–1.82	0.71
Localization 2	Apex vs. non-apex	1.97	0.98–3.51	0.05
Gleason score at biopsy (≥ 8)	< 7 vs. ≥ 8	2.56	1.53–4.26	0.0004
% positive core (≥ 30)	< 30 vs. ≥ 30%	2.57	1.51–4.57	0.0004
Clinical T stage (≥ cT2c)	< cT2c vs. ≥ cT2c	1.14	0.60–2.01	0.68
Neutrophil lymphocyte ration (≥ 2.5)	< 2.5 vs. ≥ 2.5	0.82	0.42–1.49	0.53
Operative factors				
Operation method (open)	Open vs. robot	1.71	0.68–5.73	0.28
Nerve preservation (yes)	No vs. yes	0.57	0.25–1.16	0.13
Lymph node dissection (limited)	Limited vs. standard or extended	1.21	0.70–2.03	0.49
Post-operative factors				
Bleeding (≥ 500 ml)	< 500 vs. ≥ 500 ml	1.73	0.86–3.95	0.13
Gleason score at surgery (≥ 8)	< 8 vs. ≥ 8	1.88	1.08–3.18	0.03
Pathological T stage (≥ pT3a)	< pT3a vs. ≥ pT3a	1.94	1.14–3.22	0.01
Pathological N stage (pN1)	pN0 vs. pN1	10.48	3.61–24.28	0.0002
EPE (1)	0 vs. 1	2.46	1.41–4.28	0.0002
RM (1)	0 vs. 1	2.06	1.21–3.48	0.008
ly (1)	0 vs. 1	2.25	1.29–3.82	0.005
v (1)	0 vs. 1	2.77	1.45–4.96	0.003
pn (1)	0 vs. 1	2.09	1.19–3.87	0.009
PSA nadir (< 0.1)	< 0.1 vs. ≥ 0.1 ng/ml	8.34	4.10–15.51	< 0.0001

Chi square test

Table 4 shows the predictive clinicopathological parameters analyzed using pre-treatment factors alone, by multivariate analysis. Per the table, GS at biopsy (≥ 8, HR 1.92, 95% CI 1.01–3.61, *p* < 0.05) and % positive core (≥ 30%, HR 2.85, 95% CI 1.42–6.18, *p* < 0.005) could be independent predictors of biochemical progression.

**Table 4:** Preoperative predictive factor for biochemical recurrence after radical prostatectomy

Variables	Category	Multivariate		
		HR	95% CI	*p* value
PSA at biopsy (≥ 15)	< 15 vs. ≥ 15 ng/ml	1.31	0.44–4.75	0.6421
PSA density (≥ 0.5)	< 0.5 vs. ≥ 0.5	1.20	0.34–3.38	0.7542
DRE (abnormal)	Abnormal vs. normal	1.43	0.71–2.72	0.3052
Localization 2	Apex vs. non-apex	1.54	0.67–4.18	0.3239
GS at biopsy (≥ 8)	< 7 vs. ≥ 8	1.92	1.01–3.61	0.0455
% positive core (≥ 30)	< 30 vs. ≥ 30%	2.85	1.42–6.18	0.0027

Cox proportional hazard model

**Fig. 2:**
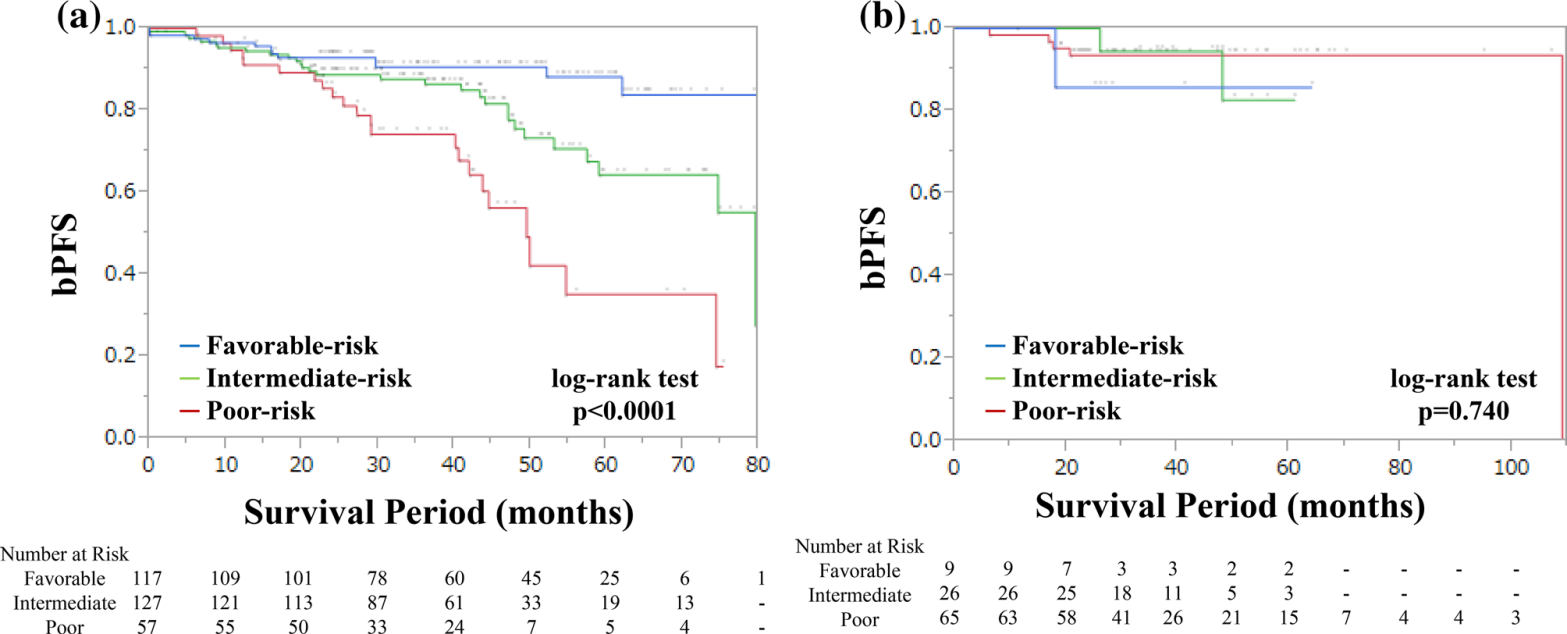
**a** Biochemical progression-free survival after RP, stratified by our risk classification model. The criteria for favorable- (0 risk factor), intermediate- (1 risk factor), and poor (2 risk factors) -risk criteria are based on the following risk factors: Gleason score at biopsy ≥ 8 and % positive core ≥ 30%. **b** Biochemical progression-free survival after IMRT stratified by our risk classification model. Favorable- (0 risk factor), intermediate- (1 risk factor), and poor- (2 risk factors) risk criteria are based on the following risk factors, Gleason score at biopsy ≥ 8 and % positive core ≥ 30%

Patients were stratified into favorable- (0 risk factor; 117 patients), intermediate- (1 risk factor; 127 patients), and poor- (2 risk factors; 57 patients) risk groups, based on the number of the following risk factors: Gleason score ≥ 8 at biopsy and % positive core ≥ 30%. Using the Cox proportional hazard model, this risk classification model could significantly predict biochemical progression after undergoing RP (favorable-risk, HR 1.0; intermediate-risk, HR 2.26; high-risk, HR 5.03; *p* < 0.0001, Fig. 2a). Two- and 5-year bPFS of the favorable-, intermediate-, and poor-risk groups were 92.9 and 88.7, 88.7 and 64.4, and 85.4 and 35.2%, respectively.

We intend to fit these criteria to the cohort of patients with high-risk PCa who were treated with IMRT. Figure 2b shows the bPFS after IMRT stratified by the risk criteria. No significant differences were observed among favorable-, intermediate-, and poor-risk groups. Two- and 5-year bPFS of the poor-risk group of the IMRT cohort were both 93.5%, respectively.

**Fig. 3:**
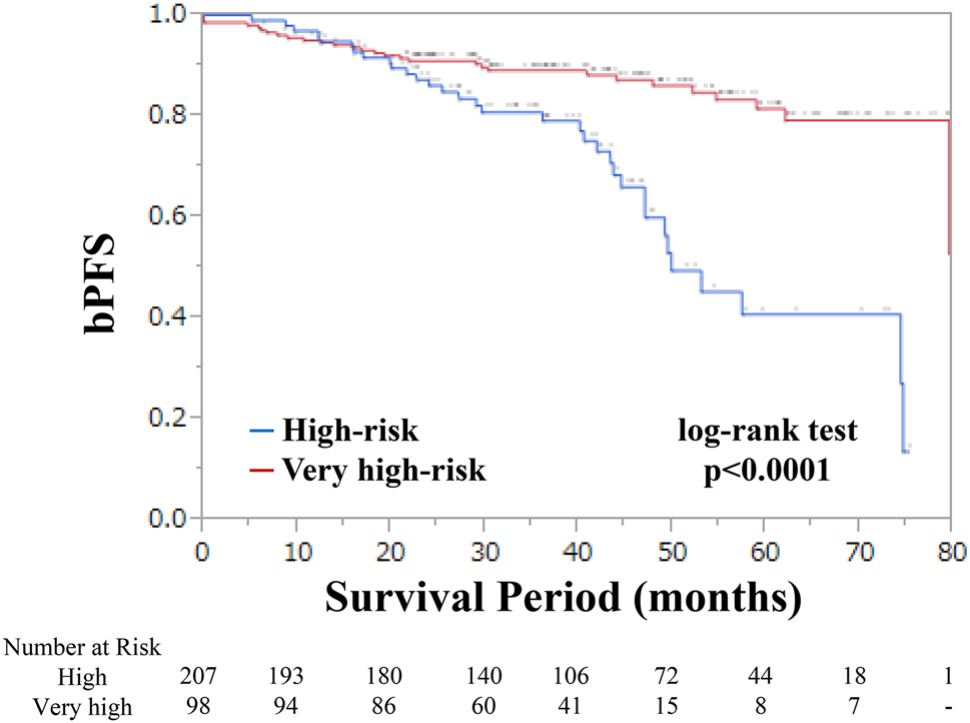
Biochemical progression-free survival after RP stratified by the risk classification model by Kobayashi et al. High-risk criteria consist of a single D’Amico high-risk factor and two low-risk factors. Very high-risk criteria consist of single D’Amico high-risk factor and at least one or more intermediate- or high-risk factors

Kobayashi et al. stratified the patients with D’Amico high-risk PCa into 2 subgroups, using D’Amico risk factors [8]. They showed a significant difference in bPFS between the high-risk subgroup (with a single D’Amico high-risk factor and 2 low-risk factors) and the very high-risk subgroup (with a single D’Amico high-risk factor and at least one or more intermediate- or high-risk factors). We stratified our patients into 2 subgroups, using the risk factors proposed by Kobayashi et al., and compared the bPFS of these subgroups (Fig. 3). There is a statistically significant difference between the subgroups (*p* < 0.0001). We calculated the c-indexes of our model and Kobayashi model in bPFS (c-index, 0.663 vs. 0.632). Based on the c-index, our risk model seems to be a little superior or comparable to the Kobayashi model in stratifying the bPFS after RP.

## Discussion

Loeb et al. reported the outcomes of 175 men with D’Amico high-risk PCa who were treated with RP without neoadjuvant and adjuvant therapy, at the Johns Hopkins hospital [9]. In their study, at 10 years after RP, bPFS was 68%, and metastatic-free and cancer-specific survivals were 84 and 92%, respectively. At 5 years after RP, the bPFS was approximately 75%. Among the high-risk criteria, a GS at biopsy ≥ 8 was the strongest independent predictor. Their data are in good agreement with our results with respect to the 5-year bPFS (70.4%), and GS at biopsy ≥ 8 as strong predictors of biochemical recurrence.

Sundi et al. analyzed the data on 1471 patients with PCa who had biochemical recurrence after RP at the Johns Hopkins hospital [10]. They concluded that the best criterion to identify early biochemical recurrence was a primary Gleason pattern of 5 or ≥ 4 cores of Gleason pattern 4 contained in biopsy specimens. Their results may support our risk stratification using GS at biopsy ≥ 8 and % positive core ≥ 30%.

The D’Amico or National Comprehensive Cancer Network criteria are frequently used to predict the outcomes of patients with high-risk PCa, which include cT2c or T3a stage, GS at biopsy ≥ 8, and PSA > 20 ng/ml; it is not known if each factor has equivalent weightage in predicting the prognoses in such patients. Walz et al. stratified the patients with high-risk PCa into 3 subgroups using 3 risk factors (≥ cT3 stage, GS ≥ 8, PSA > 20 ng/ml) [11]. They showed a significant difference in bPFS between the patients that showed 1 and 2 risk factors. Kobayashi et al. also stratified the patients with D’Amico high-risk PCa into 2 subgroups, using D’Amico risk factors [8]. As shown in Fig. 3, we stratified our patients into the subgroups using the risk factors proposed by Kobayashi et al., and compared the bPFS between the subgroups. There is a statistically significant difference between the subgroups (*p* < 0.0001). However, our risk model could be comparable to the Kobayashi model in stratifying the bPFS after RP (c-index, 0.663 vs. 0.632).

Hamada et al. investigated the pre-operative factors predicting biochemical recurrence after RP for D’Amico high-risk PCa. Based on their Cox proportional hazard regression analysis, PSA density ≥ 0.4 and percentage positive cores ≥ 70% from the dominant side may be the significant predictors of biochemical progression after RP [12]. Based on the number of the predictive factors, they stratified patients into low- (0 risk factor), intermediate- (1 risk factor), and high-risk (2 risk factors) groups. Their risk classification model could significantly predict biochemical progression after RP. They showed the important role of % positive core as a predictive factor for biochemical progression after RP.

How should we utilize our risk stratification model in the clinical setting? In our study, bPFS at 5 years after RP was nearly 90% in the favorable-risk group, which is comparable with the corresponding values in the IMRT-treated group, with the concomitant use of the 2-year ADT. Although the Japanese patients with PCa seem more tolerant to adverse effects compared to their counterparts in the Western countries, approximately 10% loss in bone mineral density during the first 1-year ADT treatment was reported in Japanese patients, too [13]. Patients classified in the favorable-risk group who were treated with RP may not experience several adverse events of ADT, without compromising the oncological outcome. In contrast, patients classified in the poor-risk group who were treated with IMRT had bPFS comparable to those of corresponding patients in the low- and intermediate-risk groups in our study. Previous reports showed the prognostic value of GS and % positive core in the patients with PCa treated by external beam radiotherapy (EBRT) and ADT. A study reporting the outcome of a high-risk group of patients treated with 78 Gy of EBRT and ADT demonstrated that GS 8–10, PSA > 20 ng/ml, and clinical stage T3 could be unfavorable parameters for bPFS and that GS was the only factor to correlate independently with cancer-specific survival [14]. And, another study showed that % positive core could be a significant predictor for bPFS in the patients with PCa treated by EBRT, independently of other known prognostic factors as cT stage, GS, and PSA. [15]. In our study, inadequate fitting of our risk model in the IMRT-treated group may be biased by large proportion of the high-risk cases and different patient characteristics in the IMRT cohort, and most importantly be influenced by the effect of 2-year androgen deprivation therapy. Different criteria of biochemical progression of RP and IMRT may also be affected. These data may help to frame appropriate treatment choices between RP and IMRT, for patients with PCa.

Our study has some limitations; this was a retrospective multicenter study without central review of pathology, and with inconsistently performed extended LND. According to the EAU guideline 2017, extended LND should be performed in all patients with high-risk PCa, as the estimated risk for positive lymph nodes in such patients is 15–40% [16]; however, only approximately 50% of the patients in our study were treated with limited LND, and the pN1 cases were only 2% involved. Nevertheless, our 5-year bPFS is identical to that obtained in the Johns Hopkins study. Current literature does not support a direct therapeutic effect of LND during RP [17].These reports suggest that the diagnoses and surgical techniques used in our high-risk cohort of patients with PCa may not deviate significantly from those used in patients in contemporary studies of high-risk PCa.

We retrospectively analyzed the clinicopathological data of the patients with D’Amico high-risk PCa who were treated with RP without neoadjuvant or adjuvant therapy to identify the patients who could be treated with RP alone. Patients were stratified into 3 risk groups using Gleason score (< 8 vs ≥ 8) at biopsy and the % positive core (< 30 vs ≥ 30%) for predicting the biochemical progression after RP.
